# Association between Metabolic Syndrome and the Musculoskeletal System

**DOI:** 10.3390/nu15204475

**Published:** 2023-10-22

**Authors:** Wenbiao Shi, Qian Zhang

**Affiliations:** Key Laboratory of Precision Nutrition and Food Quality, Department of Nutrition and Health, China Agricultural University, Beijing 100193, China; wenbiao.shi@cau.edu.cn

Metabolic syndrome (MetS) and osteoporosis are chronic health disorders worldwide. MetS is a condition characterized by clinical features like abdominal obesity, hyperglycemia, hypertension, and dyslipidemia [1]. Osteoporosis is a condition characterized by decreased bone mineral density (BMD) and increased risk of fracture, whose prevalence is growing because of the increased aging population. Each kind of MetS can affect bone metabolism in different ways, with contradictory effects and mixed conclusions, suggesting the importance of investigation and discussion of this association.

Obesity is defined as a person with a body mass index (BMI) of 30 or more, which is a result of excessive caloric intake and/or insufficient energy expenditure [2]. Obesity promotes the rate of some chronic diseases like hypertension, type 2 diabetes, cardiovascular risk, and cancer [3]. Usually, obesity is taken as a risk factor for osteoporosis. However, some studies showed that for a certain percentage of people, BMI was positively correlated with increased BMD [4,5]. The classical interpretation of this phenomenon is that higher body weight applies higher mechanical loading on the bone, thereby stimulating higher bone formation [6]. Studies found that beyond a saturated BMI, the increase in BMI outpaces the increase in BMD [7]. When the android percent fat is greater than a particular value, there is a significant negative association between adiposity measures and BMD [8]. Obesity also influences bone formation in many other different ways, like via hormone signaling such as insulin, estrogen, and adiponectin; via system inflammation; or via the lipid metabolism pathway, amongst others. According to the current studies in the literature, the relationship between obesity and bone homeostasis is complex and inconclusive, involving multiple factors and different molecular mechanisms.

Hyperglycemia is a hallmark of diabetes. Both type 1 diabetes mellitus (T1DM) and type 2 diabetes mellitus (T2DM) are associated with higher fracture risk [9]. Consistent with the fracture rate, BMD is decreased in T1DM [10]. However, BMD is unchanged or increased in T2DM, indicating decreased bone strength and quality [9]. Insulin receptor signaling regulated by glucose metabolism in osteoblast controls osteocalcin expression via the Twist2-Runx2 pathway [11]. Hyperglycemia also indirectly regulates bone formation by modulating the PTH and vitamin D systems [12].

It is well known that bone marrow-derived cells have played a role in hypertension in the last decade [13]. Interestingly, hypertension also influences osteoporosis, particularly in women. A prospective study in 3676 white elderly women showed that their yearly bone loss rate was almost doubled compared to the lowest bold pressure value to the highest one [14]. A Meta-analysis summarized 28 independent studies and 1,430,431 participants and revealed that both in Asia and Europe, the osteoporotic fracture risk is higher in people with hypertension [15]. However, compared with human studies, there are few animal studies that show the effect of hypertension on osteoporosis. One research study presented at the American Heart Association’s Hypertension 2022 Conference treated both young and old mice with angiotensin II to build a hypertension model. They observed bone loss in young mice with angiotensin II but not in the old ones [16]. Many more studies need to be conducted to uncover the link between hypertension and bone health.

Dyslipidemia (DL) refers to a condition with a wide range of abnormal plasma lipid metabolism (triglyceride, total cholesterol, low-density lipoprotein cholesterol, and high-density lipoprotein cholesterol). Osteoblasts use fatty acids as fuel sources, which are required for matrix production and mineralization at the late stages of differentiation [17]. Dyslipidemia is closely related to osteoporosis. Most of the studies showed that patients with dyslipidemia are more likely to be diagnosed with lower BMD or osteoporosis [18]. Dyslipidemia may cause atherosclerosis due to increased lipids accumulation, which also includes the process of mineral accumulation but not with the same mechanism of bone remodeling.

The studies in this Special Issue focus on the nutritional factors related to all kinds of MetS, to expand our knowledge about the association of MetS with bone metabolism. As the changes in lifestyle and global aging, the number of MetS and osteoporosis are both increasing every year. It is necessary to further explore these areas and better characterize the connection between these diseases to propose new strategies to solve the problem and help the healthcare industry make better nutritional suggestions.
